# Finite Element Analysis of Upper Airway in Ansa Cervicalis Stimulation for Obstructive Sleep Apnea

**DOI:** 10.1002/lary.70603

**Published:** 2026-05-06

**Authors:** Mukund Gupta, Songrui Li, Haoxiang Luo, David T. Kent, Yike Li

**Affiliations:** ^1^ Department of Mechanical Engineering Vanderbilt University Nashville Tennessee USA; ^2^ Department of Otolaryngology‐Head and Neck Surgery Vanderbilt University Medical Center Nashville Tennessee USA

**Keywords:** ansa cervicalis stimulation, caudal traction, finite element modeling, obstructive sleep apnea, upper airway biomechanics

## Abstract

**Objective:**

To develop a subject‐specific, three‐dimensional finite element (FE) model of the human upper airway and evaluate how ansa cervicalis stimulation (ACS), a novel neurostimulation for obstructive sleep apnea (OSA), alters upper airway anatomy under physiologic loading.

**Methods:**

Upper airway anatomy was reconstructed from a head‐and‐neck CT of an adult female using a semi‐automated pipeline, including segmentation, smoothing, tetrahedral meshing, and registration. Linear elastic material properties were assigned from the literature. ACS was simulated as a caudal load on the anterior thyroid cartilage, and inspiratory collapse tendency was mimicked with a −70 Pa luminal negative pressure. Structural displacement and cross‐sectional area (CSA) changes were quantified at the soft palate, lateral pharyngeal wall, tongue base, and epiglottis.

**Results:**

ACS produced caudal displacement of the thyroid cartilage by approximately 10 mm with coordinated motion of the hyolaryngeal complex and longitudinal pharyngeal wall strain. It also tilted the epiglottis antero–inferiorly and increased its curvature, reducing posterior–inferior motion under negative pressure by more than 50% (5.0 to 2.2 mm). Retro‐epiglottic CSA increased by 68.8% without negative pressure (42.43 to 71.63 mm^2^) and by 2980.3% with negative pressure (0.79 to 24.19 mm^2^). Retropalatal CSA improved by 34.4% without negative pressure (65.04 to 87.42 mm^2^) and by 20.3% with negative pressure (16.98 to 20.43 mm^2^).

**Conclusion:**

This proof‐of‐concept, subject‐specific FE model shows that ACS imparts caudal traction through the hyolaryngeal complex, producing multilevel anatomic stabilization under physiologic loading. The findings establish a quantitative, hypothesis‐generating framework for interrogating ACS‐mediated airway stabilization mechanisms and support further investigation across broader OSA populations and collapse phenotypes.

**Level of Evidence:**

N/A

## Graphical abstract

We developed a subject‐specific FE model to investigate how ACS stabilizes the upper airway in OSA. Simulations demonstrate that caudal traction applied through the hyolaryngeal complex produces coordinated multilevel airway stabilization, with pronounced effects at the retropalatal and retro‐epiglottic regions under physiologic loading. This proof‐of‐concept modeling framework provides mechanistic insight into ACS and supports further hypothesis‐driven investigation of neurostimulation‐based airway therapies.

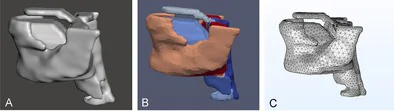

## 1 Introduction

Obstructive sleep apnea (OSA) is a common disorder affecting 10% to 30% of adults in North America, with its prevalence rising alongside the obesity epidemic [1]. Characterized by repeated episodes of upper airway obstruction during sleep, OSA is associated with an elevated risk of cardiovascular disease, diabetes, and neurocognitive impairments [2, 3, 4]. While continuous positive airway pressure (CPAP) is the standard therapy, nearly half of the patients exhibit poor adherence, leading to suboptimal outcomes [5, 6]. Alternative treatments, such as hypoglossal nerve stimulation (HNS), help selected patients but have limited efficacy and candidacy [7, 8, 9], underscoring the necessity for novel therapeutic strategies.

Ansa cervicalis stimulation (ACS), which targets the infrahyoid strap muscles, including the sternothyroid muscle, has emerged as a promising intervention [10, 11, 12, 13, 14, 15, 16]. These muscles play a crucial role in maintaining airway patency by exerting a caudal pull on the pharynx, mimicking the stabilizing effects of lung volume expansion [17, 18]. Our recent study provided physiologic evidence that ACS reduced collapsibility across all upper airway structures, with greater effects on palatal, oropharyngeal lateral wall, and epiglottic collapse than the tongue base [13]. These findings suggest that ACS and HNS have different morphological and biomechanical impacts on various upper airway structures. However, there is a paucity of data on the anatomical effect of ACS [11].

Advancements in computational biomechanics allow for the noninvasive study of human airway properties through computer modeling and other numerical techniques, particularly finite element (FE) analysis. FE models have been developed to study airway biomechanics in both healthy and pathological conditions, including OSA, as well as to evaluate the airway's response to various therapeutic interventions [19, 20, 21, 22]. These models can represent complex geometry and material behavior and can be combined with physics‐based approaches such as computational fluid dynamics to simulate airflow–tissue interactions [22, 23, 24]. These tools provide mechanistic insight into airway patency and have the potential to guide individualized treatment.

Computational modeling has been used to investigate the biomechanical effects of caudal tracheal displacement on upper airway patency, in combination with airflow dynamics and animal studies [25, 26, 27, 28]. Kairaitis and colleagues demonstrated that increasing lung volume reduces upper airway collapsibility by inducing caudal tracheal displacement, which increases longitudinal strain and decreases extraluminal tissue pressure [28]. Building on this, Amatoury et al. showed that these effects are spatially heterogeneous and mediated by the hyoid bone, which acts as a mechanical fulcrum redistributing loads across the upper airway [27]. Their subsequent FE model suggested that hyoid mobility is essential for transmitting mechanical forces to surrounding tissues and enhancing airway patency [25]. However, these studies were limited to animal models with simplified two‐dimensional geometries and did not resolve the distinct responses of individual upper airway structures. Moreover, the methods used to induce caudal traction, such as lung volume manipulation or direct tracheal displacement, target different anatomical pathways than ACS, which applies caudal force to hyolaryngeal structures [10]. These limitations highlight the need for a high‐resolution, anatomically detailed FE model of the human upper airway to investigate the specific biomechanical mechanisms of ACS.

In this proof‐of‐concept study, we develop a subject‐specific, three‐dimensional (3D) FE model to assess the biomechanical effects of ACS on the upper airway structures under physiologic loading conditions. The primary objective is to establish a computational framework for elucidating tissue deformation patterns and force‐transmission mechanisms through which ACS may enhance airway stability in OSA. We anticipate that this model will enable generation of testable hypotheses regarding ACS‐mediated airway stabilization and provide a foundation for future studies extending to broader OSA populations and collapse phenotypes.

## 2 Materials and Methods

### 2.1 Data Acquisition

This study was approved by the Institutional Review Board at Vanderbilt University Medical Center (Protocol #212305). Patient consent was not required due to the retrospective design and use of de‐identified public data. A head and neck CT scan of a 43‐year‐old female patient undergoing thyroid exams was obtained from the Imaging Data Commons, a public database of the National Institutes of Health [29]. Patient diagnosis and history were unavailable, but all upper airway structures appeared radiologically normal. The scan, performed using a SOMATOM Sensation 64 CT scanner (Siemens Inc.), spanned from the top of the skull to approximately the 7th thoracic vertebra. Imaging parameters included a section thickness of 3 mm, a field of view of 251 × 251 mm, a pixel spacing of 0.49 mm, and a window setting centered at 70 HU with a width of 410 HU. The images were stored in DICOM format.

### 2.2 Segmentation and Mesh Generation

**FIGURE 1:**
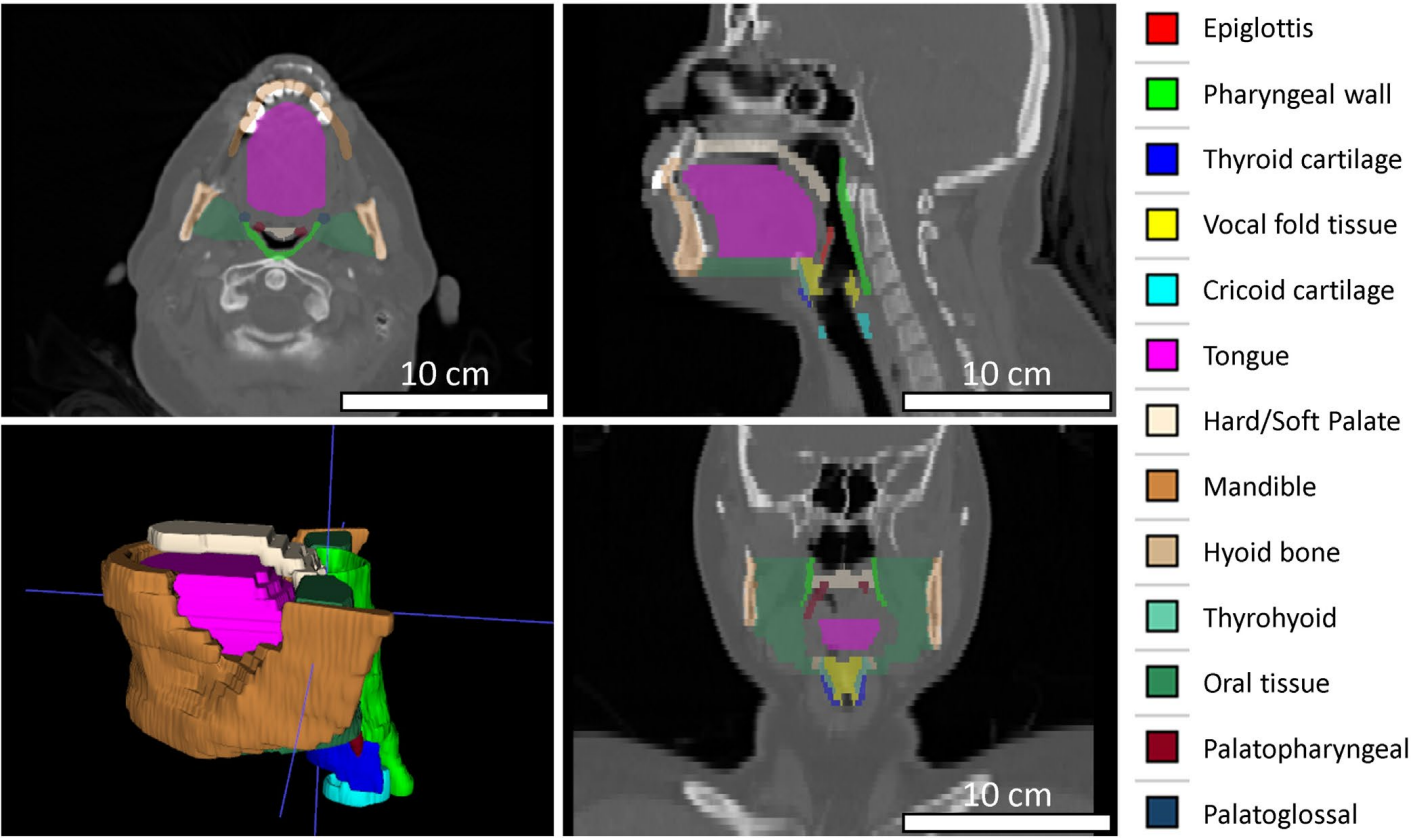
The segmentation process in ITK‐SNAP showing different anatomical structures labeled with various colors.

**FIGURE 2:**
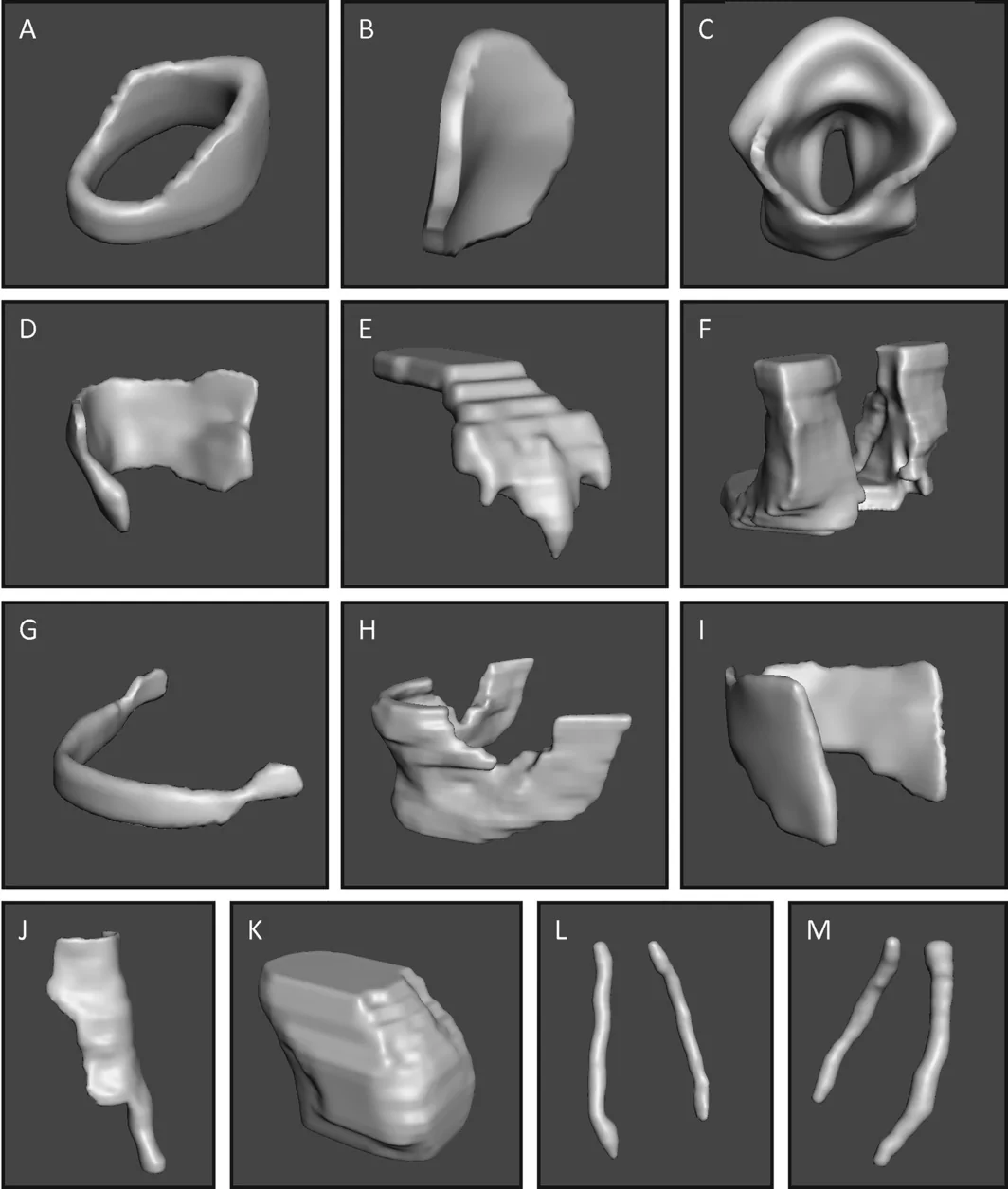
Smoothed anatomical components. (A) cricoid cartilage, (B) epiglottis, (C) vocal fold tissue, (D) thyrohyoid membrane, (E) hard and soft palate, (F) soft tissue in the oral cavity, (G) hyoid bone, (H) mandible, (I) thyroid cartilage, (J) pharyngeal wall, (K) tongue, (L) palatopharyngeus muscles, (M) palatoglossus muscles.

Manual segmentation was performed in ITK‐SNAP (Version 4.2.0) to label key anatomical structures of the upper airway, including the tongue, epiglottis, soft palate, mandible, pharyngeal wall, cricoid cartilage, vocal fold, hyoid bone, thyroid cartilage, infrahyoid muscles, palatoglossus muscles, palatopharyngeus muscles, and the connective tissue between the mandible and the pharyngeal wall (Figure 1). The segmented surfaces were exported to AutoDesk Meshmixer (Version 3.5.0) for smoothing to remove uneven features (Figure S1A,B). Each smoothed component was subsequently exported as the .stl format to COMSOL Multiphysics (Version 6.0) to generate an unstructured volumetric mesh consisting of tetrahedral elements (Figure S1C). The resulting COMSOL mesh was exported as a Nastran (.nas) file. This process was repeated for all upper airway structures (Figure 2).

**FIGURE 3:**
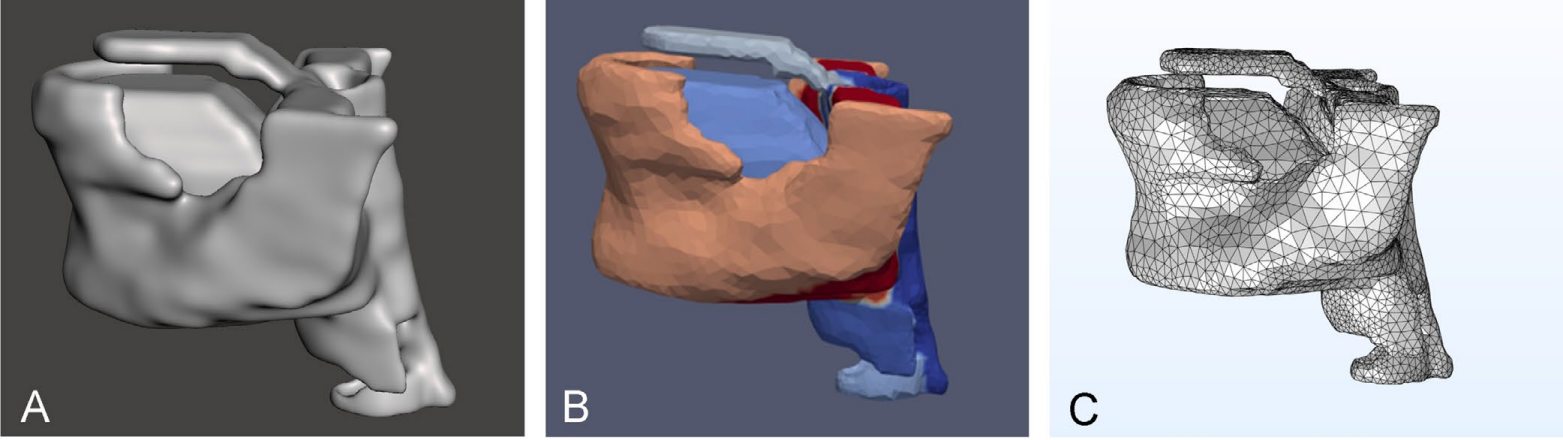
Unified body mesh of the upper airway. (A) unlabeled structure, (B) labeled components, and (C) mesh elements.

To address mismatches between individual meshed structures at their interfaces, a unified, unlabeled segmentation was created by combining all the components in ITK‐SNAP. This unified segment was smoothed in Meshmixer (Figure 3A) and converted into a volumetric mesh of tetrahedral elements in COMSOL, following the same steps as for the individual structures. A custom MATLAB code was developed to read the mesh data from multiple Nastran files and perform registration. During this process, all components were identified and flagged on the unified mesh by comparing the whole unit with individual meshes (Figure 3B). The tetrahedral elements were assigned to their respective components, and the labeled unified mesh was imported into COMSOL as the FE mesh (Figure 3C).

### 2.3 Tissue Properties and Boundary Conditions

Material properties for the labeled components on the unified mesh were assigned based on literature (Table S1). For simplicity in this proof‐of‐concept model, we set all components to be linear elastic. To further assess the validity of this assumption, we tested a hyperelastic, nonlinear model for the tongue, which exhibits large deformations in previous research [30, 31], and the results remained consistent. Additionally, the Poisson ratio of the tongue was set at 0.49, approximating incompressibility and reflecting its muscular hydrostat property. The boundary conditions applied included fixed constraints, prescribed boundary loads, and displacement constraints. The hard palate and mandible were fixed to provide support and anchoring for the entire structure (Figure S2A–C). The contraction of the sternothyroid muscle was modeled as a 1 N boundary load on the bottom edge of the anterior surface of the thyroid cartilage, directed toward the negative z‐axis (Figure S2A). The cricoid cartilage was constrained to move only in the z‐direction and fixed along the x‐ and y‐directions to account for potential sideways movement caused by minor asymmetry in the cricoid cartilage reconstruction. Additionally, the pharyngeal wall movement was constrained so that it could only move along the direction of the spine.

### 2.4 Outcome Assessments Under Physiologic Loading

**FIGURE 4:**
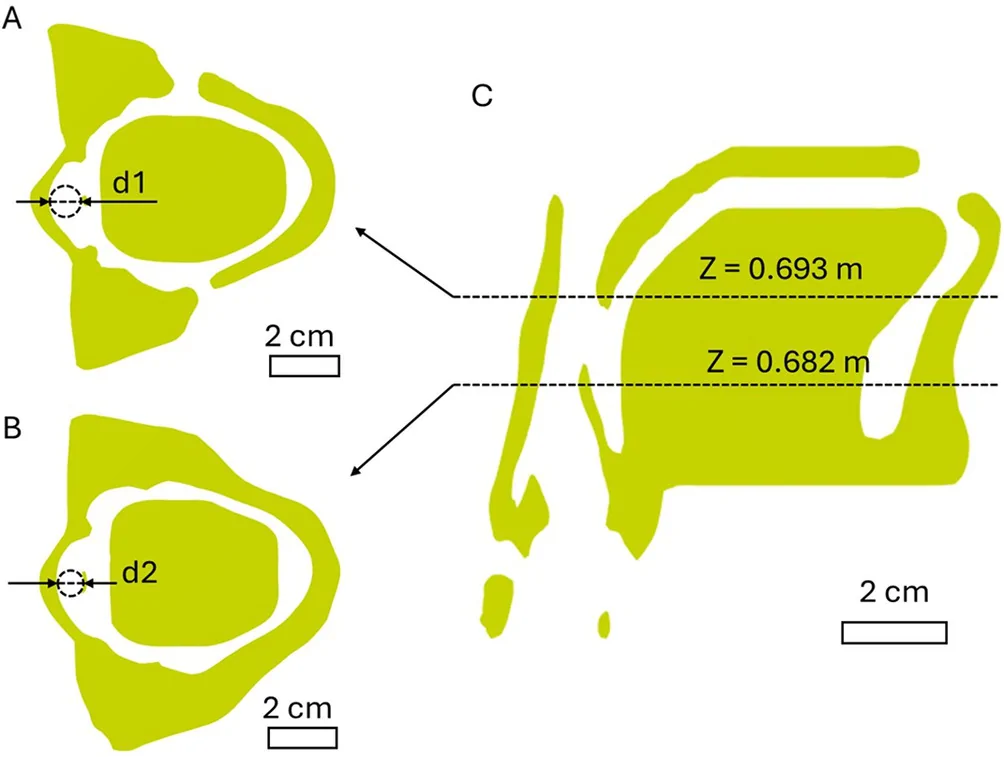
Axial and sagittal cross‐sections used for area measurement. (A, B) Axial (xy‐plane) cross‐sections at the soft palate and epiglottis, with airway lumen area quantified by the largest inscribed circle (*d*1, *d*2). (C) Mid‐sagittal (yz‐plane) view indicating the corresponding slice locations.

To mimic inspiratory conditions that drive collapse in OSA, a −70 Pa negative pressure was applied uniformly as a quasi‐static load to the posterior surfaces of the epiglottis, soft palate, tongue base, as well as the luminal surface of the pharyngeal wall (Figure S2D). Four loading conditions were evaluated: rest (no ACS, no negative pressure), ACS only, negative pressure only, and ACS + negative pressure. Airway CSA was assessed by computing the largest inscribed circles on two axial slices, including retropalatal and retro‐epiglottic levels (Figure 4). This metric provides a conservative, reproducible estimate of the flow‐limiting dimension and is less sensitive to irregular lumen boundaries and segmentation variability than full‐contour area measurements.

## 3 Results

### 3.1 Thyroid Cartilage

**FIGURE 5:**
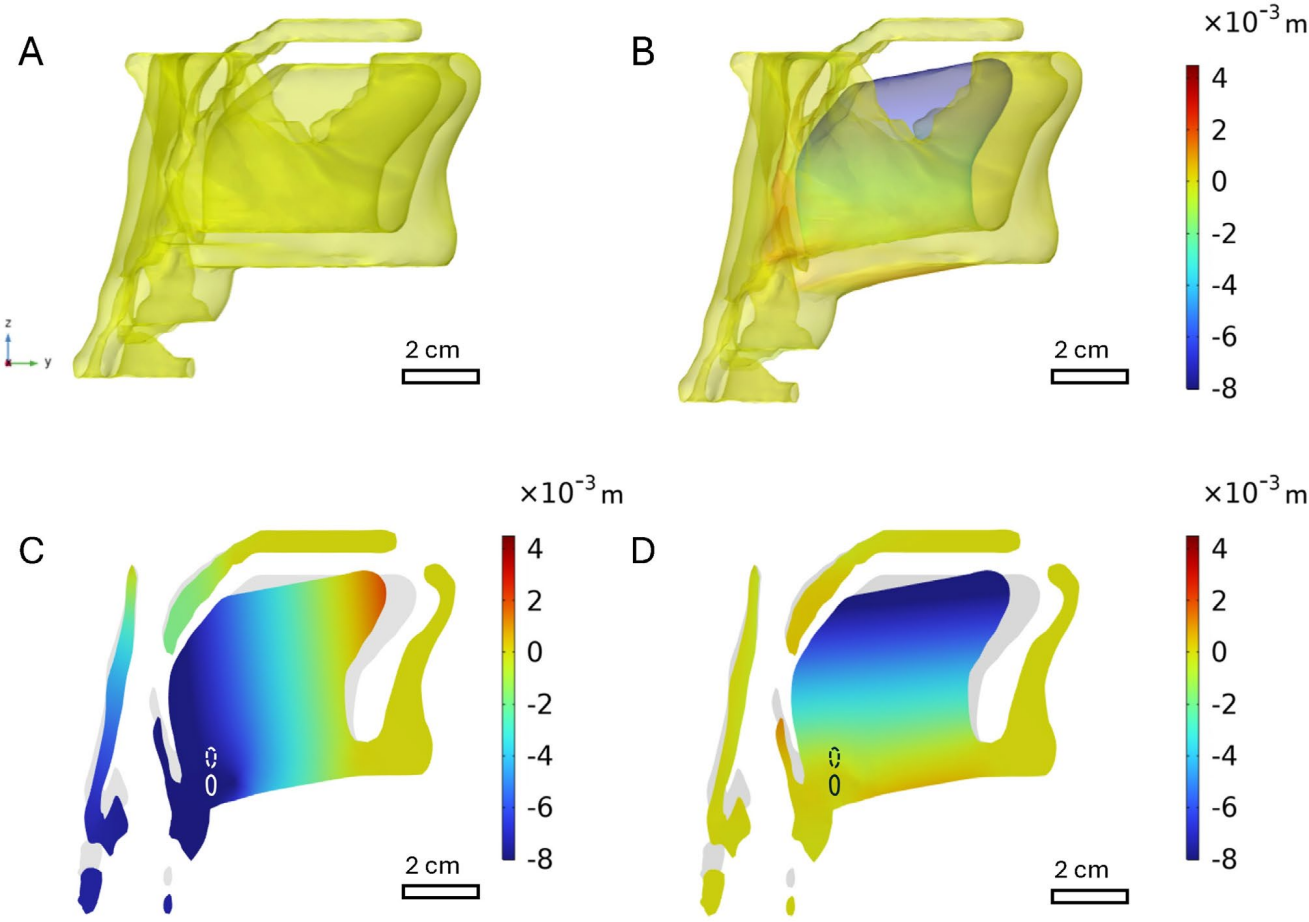
Effect of ACS on upper airway structures in the absence of negative pressure. (A) Baseline 3D view; (B) ACS 3D view colored by the y‐displacement; (C) ACS sagittal (yz‐plane) view colored by the z‐displacement; (D) ACS sagittal (yz‐plane) view colored by the y‐displacement; Gray contours in (C) and (D) denote the baseline upper airway conformation. The dashed and solid circles in (C) and (D) indicate the initial and displaced positions of the hyoid bone, respectively.

ACS activated the sternothyroid muscles, exerting a direct caudal (downward) pull on the anterior surface of the thyroid cartilage. This force displaced the thyroid cartilage inferiorly by up to 10 mm with an accompanying anteroinferior tilt. Coupled with this motion, the soft tissues beneath the mandible, the hyoid, the epiglottic base, and the posterior tongue base moved downward by approximately 5–10 mm (Figure 5).

### 3.2 Tongue

**FIGURE 6:**
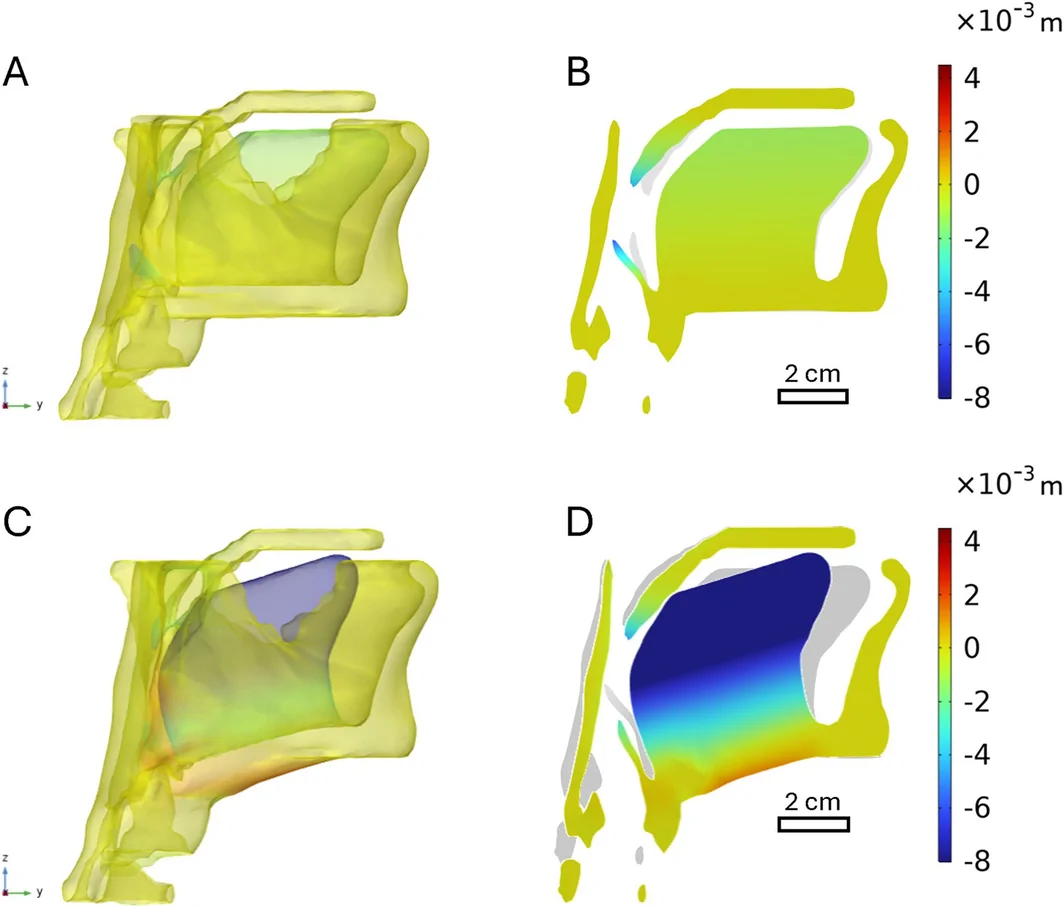
Upper airway deformation under negative pressure, with and without ACS. (A, B) Negative pressure alone: (A) 3D view; (B) sagittal (yz‐plane) view. Gray contours indicate the baseline conformation (no ACS, no negative pressure); colors represent y‐displacement. (C, D) Negative pressure with ACS: (C) 3D view; (D) sagittal (yz‐plane) view. Gray contours indicate the conformation under negative pressure without ACS; colors represent y‐displacement.

The resulting tension from the thyroid cartilage displacement pulled the tongue base down by 7 mm through soft tissue connections, producing a modest upward pitch of the tongue body toward the palate. It minimally changed the retroglossal space but moved the posterior tongue inferiorly without contacting the soft palate (Figure 5). This potentially reduced palatal loading from the tongue. ACS effects at the tongue base were preserved under negative pressure, with minimal additional displacement (Figure 6).

### 3.3 Pharyngeal Wall

Caudal traction induced elongation of the pharyngeal wall in the direction of the trachea, with maximum displacement of approximately 9 mm near the thyroid cartilage (Figure 5). This longitudinal strain is consistent with increased wall stiffness, akin to tensioning an elastic band, thereby reducing susceptibility to collapse [24, 32].

### 3.4 Epiglottis

**FIGURE 7:**
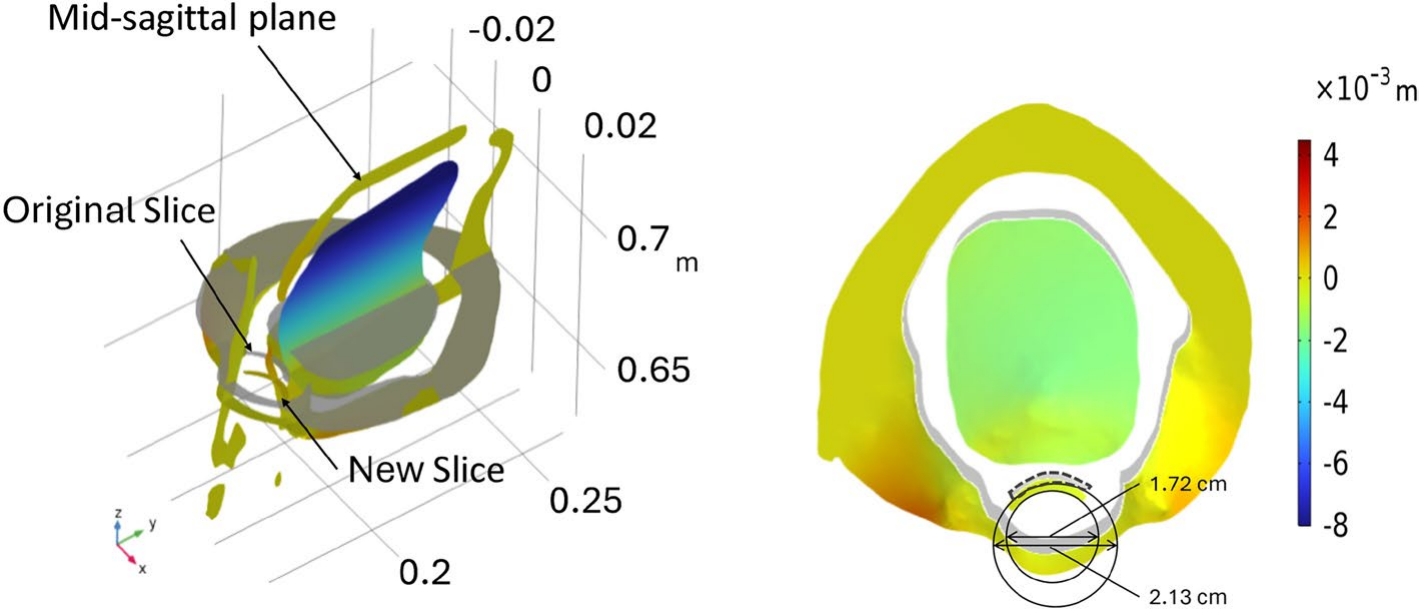
Effect of ACS on the epiglottis. (Left) 3D view showing the original and ACS positions of an axial slice through the epiglottis. ACS produces inferior (−z‐axis) displacement of the slice. (Right) Axial slice illustrating increased epiglottic curvature with ACS, demonstrated by two inscribed circles for the epiglottis with and without ACS; the dashed contour depicts the epiglottis under negative pressure without ACS. Colors denote y‐displacement.

ACS shifted the epiglottis anteriorly by 1 mm and inferiorly by 9 mm, tilting it more upright and away from the posterior pharyngeal wall and glottis (Figure 5). The nonuniform displacement increased epiglottic curvature, suggesting geometric stiffening (e.g., similar to how a curved tape measure maintains shape when extended) despite unchanged material properties and thereby greater mechanical stability and resistance to collapse (Figure 7).

Under negative pressure alone, the epiglottis exhibited a posterior–inferior displacement of 5 mm, substantially narrowing the supraglottic airway (Figure 6A,B). In contrast, ACS counteracted this by generating a forward tilt and reducing the posterior displacement to 2.2 mm (56.0% improvement), reversing the restriction (Figure 6C,D). Correspondingly, retro‐epiglottic CSA increased by 68.82% without negative pressure (42.43 to 71.63 mm^2^) and by 2980.25% with negative pressure (0.79 to 24.19 mm^2^, Table 1), indicating both repositioning and shape‐related mechanical stabilization of the airway.

**TABLE 1:** Cross‐sectional area measurement at the retro‐epiglottic and retropalatal regions under varying conditions. Percentages represent changes relative to the baseline within the same pressure condition.

	No negative pressure			With negative pressure		
	Baseline	ACS	% Change	Baseline	ACS	% Change
Retro‐epiglottic CSA (mm^2^)	42.43	71.63	68.82%	0.79	24.19	2980.25%
Retropalatal CSA (mm^2^)	65.04	87.42	34.41%	16.98	20.43	20.29%

Abbreviations: ACS, ansa cervicalis stimulation; CSA, cross‐sectional area.

### 3.5 Soft Palate

With ACS, the soft palate moved anteriorly by 0.5 mm and inferiorly by 2 mm, primarily via transmission through the palatopharyngeus linking the thyroid cartilage and palate. Negative pressure alone shifted the soft palate posteriorly by approximately 4 mm, narrowing the retropalatal airway. ACS partially restored the soft palate position by about 1 mm, and with the tongue base repositioned, likely reduced palatal loading from the tongue (Figures 5 and 6). These deformations yielded a retropalatal CSA increase of 34.41% without negative pressure (65.04 to 87.42 mm^2^) and 20.29% with negative pressure (16.98 to 20.43 mm^2^, Table 1).

## 4 Discussion

This study presents an anatomy‐derived, CT‐based FE model to simulate the effects of ACS on upper airway structures. Using a semi‐automatic geometry‐processing pipeline, the model reconstructed a unified, labeled airway and showed that ACS generates coordinated caudal motion and deformation across multiple structures, substantially increases retropalatal and retro‐epiglottic CSA, and mitigates airway narrowing under negative pressure. These anatomical effects were consistent with our clinical observations and physiological data [10, 11, 12, 13, 14, 16, 33], supporting the feasibility of using FE modeling to simulate upper airway responses to ACS. Collectively, the findings strengthen the traction‐based mechanism by which ACS stabilizes the airway and suggest that this modeling approach may help predict treatment response.

FE analysis has been utilized in previous upper airway and OSA research, including in patients undergoing HNS and other treatments [19, 20, 21, 22, 34, 35, 36]. Within this context, our work provides an early computational investigation of ACS in a human anatomy‐derived configuration, explicitly linking thyroid‐cartilage traction to multilevel airway responses. The methodology established in this study effectively captures individual anatomical features for upper airway reconstruction and produces an integrated mesh with properly identified, connected components, in contrast to analyses of isolated, disconnected parts [35, 36, 37]. This unified body enables realistic transmission of loads and constraints across tissues and simplifies subsequent modifications as the model evolves, that components can be conveniently added, removed, or modified through a modular pipeline. In addition, the approach is compatible with incorporating higher‐resolution and complementary imaging (e.g., magnetic resonance imaging, optical coherence tomography) and multimodal data to refine segmentation and material assignment. These features position the model for ongoing research as new evidence on OSA and ACS emerges and for mechanistic investigation of other novel OSA treatments.

Our findings align with and extend prior experimental and computational studies on caudal tracheal traction. Previous work has highlighted the critical role of the hyoid bone as a load‐redistribution hub, demonstrating that its mobility is essential for transmitting stabilizing forces [25, 27]. The present model reproduces these traction‐based principles within a human anatomy‐derived geometry, but via the ACS pathway where force is applied through the anterior thyroid cartilage rather than the trachea. Coordinated longitudinal hyoid motion with surrounding soft tissues transmits caudal displacement to the other pharyngeal structures, yielding deformation patterns such as pharyngeal wall elongation that are consistent with prior physiologic and imaging studies of sternothyroid‐mediated laryngeal descent [38, 39]. Importantly, whereas previous models often used simplified two‐dimensional or nonhuman geometries that did not resolve structure‐specific responses [21, 25, 26, 27], our analysis quantifies regional effects across the soft palate, lateral pharyngeal wall, tongue base, and epiglottis—sites central to contemporary OSA phenotyping [21, 25, 26, 27]. A notable addition is the observation that ACS increases epiglottic curvature, suggesting geometric stiffening despite unchanged material properties. This shape‐related stabilization provides a plausible explanation for the large retro‐epiglottic CSA gains and the mitigation of posterior–inferior motion under negative pressure, adding a complementary mechanism to the established caudal‐traction narrative.

The modeling results are also consistent with our recent human physiology findings, strengthening a coherent mechanistic picture for ACS [13]. Breath‐by‐breath assessments revealed that ACS reduced collapsibility across sites, with greater ventilatory benefit at the soft palate, lateral walls, and epiglottis than at the tongue base. This FE model recapitulates this structure‐specific pattern: inferior displacement of the hyolaryngeal complex only modestly altered the retroglossal space, whereas retropalatal and retro‐epiglottic CSA increased substantially. This provides corroborating evidence to airflow physiology and endoscopic observations, connecting geometric analysis with functional and visual assessments and suggesting that ACS relieves palatal loading by repositioning the tongue base away from the soft palate, increases longitudinal strain to stiffen the pharyngeal wall, and stabilizes the epiglottis via repositioning and curvature‐related geometry. These converging lines of evidence support the clinical rationale that ACS may be particularly effective for epiglottic, lateral wall and palatal collapse phenotypes, and potentially complementary to HNS in mixed‐site obstruction.

When further contextualizing our ACS findings alongside prior HNS data, both convergent and divergent patterns emerge, suggesting partially overlapping yet mechanistically distinct modes of airway modulation. Previous HNS studies consistently demonstrate multilevel airway enlargement, with robust effects at the retrolingual level and more variable responses at the retropalatal airway. Human endoscopic studies report retropalatal CSA increases of approximately 50%–60% during wakefulness and up to 180% under drug‐induced sedation, with retrolingual enlargement of similar or greater magnitude [40, 41, 42]. Fluoroscopic imaging further demonstrates anterior–posterior retropalatal opening on the order of several millimeters when present, although this response occurs only in a subset of subjects [42]. In comparison, the magnitude of retropalatal enlargement predicted by the present ACS model falls within the range reported for clinically effective HNS, while producing consistent stabilization of the retro‐epiglottic airway. These differences likely reflect distinct mechanisms of force transmission, that HNS primarily advances the tongue and indirectly influences palatal position, whereas ACS applies longitudinal traction through the hyolaryngeal complex, stabilizing epiglottic and palatal regions without reliance on tongue protrusion or tongue shape modulation [40, 43, 44]. Together, these findings suggest that ACS and HNS exert complementary anatomical effects, with overlapping magnitudes of airway enlargement but distinct, site‐specific profiles of action.

As a proof‐of‐concept study aimed at establishing a computational framework for mechanistic exploration, the simulations were conducted using a limited set of modeling assumptions, including loading conditions and airway caliber metrics. These choices, however, were not arbitrary but anchored to available human physiologic and clinical evidence. The magnitude of hyolaryngeal descent predicted by the model is consistent with prior human studies demonstrating that sternothyroid and related infrahyoid muscle activation can produce inferior displacement of the hyolaryngeal complex on the order of approximately 5–15 mm [38, 45], supporting the plausibility of the selected 1 N ACS load. Likewise, the −70 Pa inspiratory pressure was chosen to represent a physiologically relevant, modest collapsing load, consistent with computational and experimental studies of upper‐airway mechanics as well as clinical observations indicating that non‐flow‐limited to mildly flow‐limited breathing typically occurs within inspiratory pressure ranges of approximately −50 to −150 Pa [46]. In addition, airway caliber was quantified using the largest inscribed circle rather than full lumen area, prioritizing a standardized and reproducible measure of the flow‐limiting dimension over detailed boundary irregularities. Together, these settings enable controlled, physiologically grounded comparisons across loading conditions and support a robust, reproducible analytic framework for interrogating airway stabilization mechanisms.

We acknowledge several limitations in this study. First, the model is derived from a single CT scan of an adult subject without a confirmed OSA diagnosis. Because this work is a proof‐of‐concept focused on the primary caudal traction mechanism, we expect the core effect to be broadly applicable. Nevertheless, generalizability should be evaluated across diverse airway morphologies and collapse phenotypes in larger OSA cohorts. Second, the CT was likely acquired during wakefulness, which may not reflect sleep‐state collapsibility due to higher muscle tone. This could understate tongue‐palate interactions in the absence of direct contact and thus the modeled palatal response. Future studies should prioritize imaging during natural sleep to better replicate physiologic conditions. Third, routine clinical CT provides limited soft‐tissue contrast and spatial resolution, potentially reducing segmentation fidelity for small connective elements. Incorporating higher‐resolution imaging modalities, such as thin‐slice CT or magnetic resonance imaging, could improve mesh accuracy and anatomical fidelity. Finally, several simplifying assumptions were adopted across the modeling framework, including loading conditions, airway caliber metrics, material properties, and boundary constraints, to establish a controlled, reproducible, and hypothesis‐generating analytic pipeline. While these assumptions are physiologically plausible and appropriate for an initial mechanistic investigation, they necessarily limit the precision of quantitative predictions for complex tissue deformation and nonlinear responses, especially for the tongue [31, 47, 48], which exhibited an unverified upward tilt in the model. Future work should incorporate more complete nonlinear and viscoelastic tissue formulations, along with systematic sensitivity analyses and expanded loading and boundary‐condition scenarios, to enhance the fidelity and predictive scope of the simulations.

Despite these limitations, our model effectively simulated the major effects of ACS on key upper airway structures, providing valuable data and a useful tool for future research. Future studies should collect more data from OSA patients during sleep and refine the model with greater anatomical detail and complexity. The ultimate goal is to improve our understanding of upper airway biomechanics and enable personalized treatment for OSA patients.

## 5 Conclusion

This proof‐of‐concept, subject‐specific FE model demonstrates that ACS applies caudal traction to the thyroid cartilage, redistributes loads through the hyolaryngeal complex, and achieves multilevel mechanical stabilization of the pharyngeal airway under physiologic loading. The modeled deformation patterns align with existing physiological data and clinical observations, supporting the plausibility of the proposed ACS mechanism and providing a quantitative framework for interrogating airway stabilization pathways. Future work incorporating OSA‐specific validation across broader patient populations and collapse phenotypes, along with patient‐specific anatomy, tissue properties, and sleep‐state imaging, will be essential to extend this framework toward hypothesis‐driven evaluation and individualized treatment strategies for OSA.

## Funding

This work was supported by the National Institutes of Health (R01HL161635).

## Disclosure

All figures and tables in this manuscript are original and created by the authors.

## Ethics Statement

This study was approved by the Institutional Review Board at Vanderbilt University Medical Center (Protocol #212305). Patient consent was not required due to the retrospective design and use of publicly available, de‐identified data.

## Conflicts of Interest

D.T.K. has received research support from Invicta Medical, Inspire Medical System, and Nyxoah SA, is a consultant to Invicta Medical and Nyxoah SA, is a scientific advisory board member with Nyxoah SA, and is an inventor on patent pending PCT/US2020/021359 and related work licensed by Nyxoah SA that is relevant to this work. The other authors declare no conflicts of interest.

## Supporting information


**Table S1:** Material properties for all components in the model.
**Figure S1:** Mesh processing steps for the pharyngeal wall. (A) Segmented, pre‐smoothing structure exported from ITK‐SNAP; (B) smoothed structure in Meshmixer; (C) final mesh generated in COMSOL.
**Figure S2:** Mechanical and aerodynamic settings in the model. (A–C) Boundary conditions assigned to various anatomical structures in the model. (D) Illustration of negative pressure applied to the upper airway.

## Data Availability

The images used in this study were obtained from publicly available imaging resources (Imaging Data Commons, NIH). Processed mesh files and analysis scripts are available from the corresponding authors upon reasonable request.
