# Supplementary material for: Finite Element Analysis of Upper Airway in Ansa Cervicalis Stimulation for Obstructive Sleep Apnea
Source: Laryngoscope. 2026 May 6;136(10):4575–84. doi: 10.1002/lary.70603 (PMC13569699; doi:10.1002/lary.70603)
Supplement: Supplementary file 1 [file LARY-136-4575-s001.pdf]

Supplementary Materials

for

Finite Element Analysis of Upper Airway in Ansa Cervicalis Stimulation for Obstructive Sleep Apnea

Table of Contents

SUPPLEMENTARY TABLES **2**

Supplementary Table 1 2

SUPPLEMENTARY FIGURES **3**

Supplementary Figure 1 3

Supplementary Figure 2 4

SUPPLEMENTARY REFERENCES **5**

SUPPLEMENTARY TABLES

Supplementary Table 1

Material properties for all components in the model.

Component	Density (kg/m^3)	Youngs Modulus (Pa)	Poisson's Ratio	Reference
**Mandible**	1680	15000000000	0.4	Duck[1], Pelteret et. al.[2]
**Pharyngeal wall**	1040	5417.4	0.49	Duck[1], Liu et. al.[3]
**Tongue**	1040	15000	0.49	Payan et. al.[4]
**Epiglottis**	1100	31942	0.3	Duck[1], Liu et. al.[3]
**Cricoid**	1100	1068666	0.29	Duck[1], Pauken et. al.[5]
**Vocal cord**	1029.9	30000	0.49	Alipour et. al.[6]
**Hyoid bone**	1900	15000000000	0.4	Pelteret[2]
**Thyroid**	1000	3000000	0.42	Pelteret[2]
**Soft palate**	1040	5520	0.499	Ayyalasomayajula et. al.[7]
**Oral tissue**	916	3346.8	0.49	Duck[1], Liu et. al.[3]
**Thyrohyoid muscle**	1041	18570	0.499	Duck[1], Liu et. al.[8]
**Palato muscle**	1041	18570	0.499	Duck[1], Liu et. al.[8]

SUPPLEMENTARY FIGURES

Supplementary Figure 1

Mesh processing steps for the pharyngeal wall. (A) Segmented, pre-smoothing structure exported from ITK-SNAP; (B) Smoothed structure in Meshmixer; (C) Final mesh generated in COMSOL.

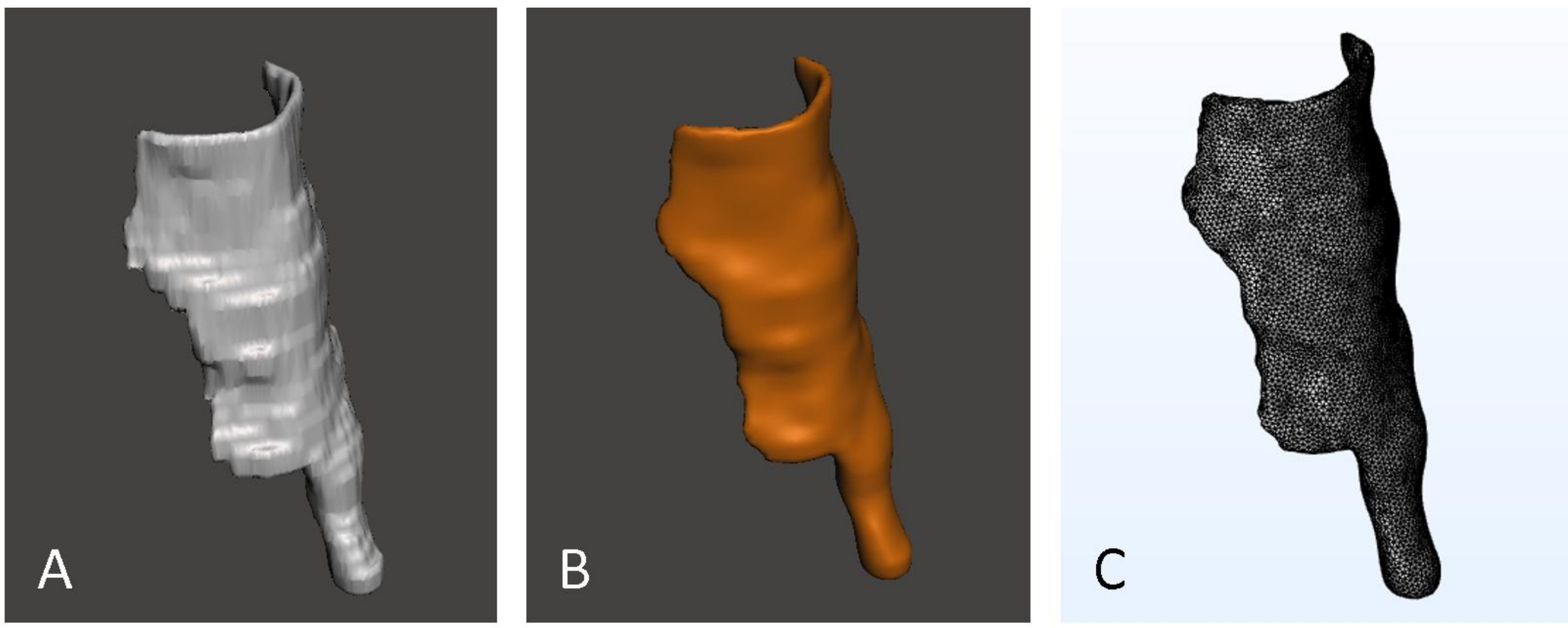

Supplementary Figure 2

Mechanical and aerodynamic settings in the model. (A-C) Boundary conditions assigned to various anatomical structures in the model. (D) Illustration of negative pressure applied to the upper airway.

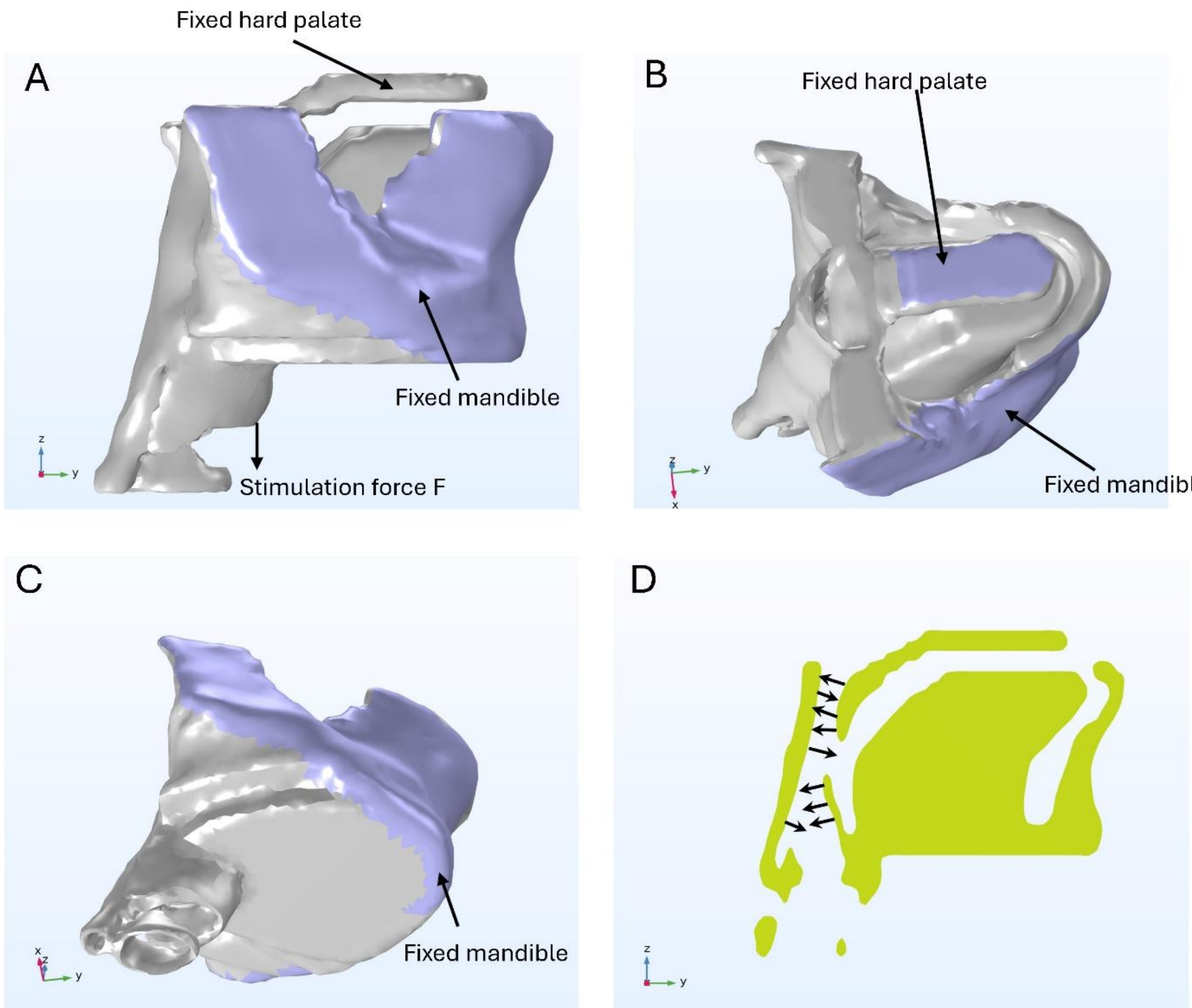

SUPPLEMENTARY REFERENCES

1. Duck FA. *Physical Properties of Tissues: A Comprehensive Reference Book*. Academic press; 1990. doi:10.1016/C2009-0-02755-X

2. Pelteret JPV, Reddy BD. Computational model of soft tissues in the human upper airway. *Int J Numer Method Biomed Eng*. 2012;28(1):111-132. doi:10.1002/CNM.1487

3. Liu Y. Fluid Structure Interaction Study of the Obstructive Sleep Apnea and Surgical Treatment Using Suture-Patch Device on the Patient’s Tongue. *UNLV Theses, Dissertations, Professional Papers, and Capstones*. Published online December 15, 2018. doi:10.34917/14279650

4. Payan Y, Bettega G, Raphaël B. A biomechanical model of the human tongue and its clinical implications. *Lecture Notes in Computer Science (including subseries Lecture Notes in Artificial Intelligence and Lecture Notes in Bioinformatics)*. 1998;1496:688-695. doi:10.1007/BFB0056255

5. Pauken CM, Heyes R, Lott DG. Mechanical, Cellular, and Proteomic Properties of Laryngotracheal Cartilage. *Cartilage*. 2019;10(3):321-328. doi:10.1177/1947603517749921

6. Alipour F, Vigmostad S. Measurement of vocal folds elastic properties for continuum modeling. *Journal of Voice*. 2012;26(6):816.e21-816.e29. doi:10.1016/j.jvoice.2012.04.010

7. Ayyalasomayajula V, Moxness M, Skallerud B. Potential of computational models in personalized treatment of obstructive sleep apnea: a patient-specific partial 3D finite element study. *Biomech Model Mechanobiol*. 2024;23(2):507-524. doi:10.1007/S10237-023-01788-1

8. Liu H, Prot VE, Skallerud BH. Soft palate muscle activation: a modeling approach for improved understanding of obstructive sleep apnea. *Biomech Model Mechanobiol*. 2019;18(3):531-546. doi:10.1007/S10237-018-1100-1
